# Study of Alzheimer’s Disease-Related Biophysical Kinetics with a Microslit-Embedded Cantilever Sensor in a Liquid Environment

**DOI:** 10.3390/s17081819

**Published:** 2017-08-07

**Authors:** Myung-Sic Chae, Jinsik Kim, Yong Kyoung Yoo, Jeong Hoon Lee, Tae Geun Kim, Kyo Seon Hwang

**Affiliations:** 1 Department of Clinical Pharmacology and Therapeutics, College of Medicine, Kyung Hee University, Seoul 02447, Korea; bechu88@gmail.com; 2 School of Electrical Engineering, Korea University, Seoul 02841, Korea; tgkim1@korea.ac.kr; 3 Department of Medical Biotechnology, College of Life Science and Biotechnology, Dongguk University, Seoul 10326, Korea; lookup2@dongguk.edu; 4 Department of Electrical Engineering, Kwangwoon University, Seoul 01897, Korea; yongkyoung0108@gmail.com (Y.K.Y.); jhlee@kw.ac.kr (J.H.L.)

**Keywords:** cantilever sensor, biosensor, viscous damping, Alzheimer’s disease

## Abstract

A microsized slit-embedded cantilever sensor (slit cantilever) was fabricated and evaluated as a biosensing platform in a liquid environment. In order to minimize the degradation caused by viscous damping, a 300 × 100 µm^2^ (length × width) sized cantilever was released by a 5 µm gap-surrounding and vibrated by an internal piezoelectric-driven self-actuator. Owing to the structure, when the single side of the slit cantilever was exposed to liquid a significant quality factor (Q = 35) could be achieved. To assess the sensing performance, the slit cantilever was exploited to study the biophysical kinetics related to Aβ peptide. First, the quantification of Aβ peptide with a concentration of 10 pg/mL to 1 μg/mL was performed. The resonant responses exhibited a dynamic range from 100 pg/mL to 100 ng/mL (−56.5 to −774 ΔHz) and a dissociation constant (K_D_) of binding affinity was calculated as 1.75 nM. Finally, the Aβ self-aggregation associated with AD pathogenesis was monitored by adding monomeric Aβ peptides. As the concentration of added analyte increased from 100 ng/mL to 10 µg/mL, both the frequency shift values (−813 to −1804 ΔHz) and associate time constant increased. These results showed the excellent sensing performance of the slit cantilever overcoming a major drawback in liquid environments to become a promising diagnostic tool candidate.

## 1. Introduction

The need exists for a rapid and accurate method for early diagnosis of Alzheimer’s disease (AD), a severe neurodegenerative disorder. Early diagnosis of AD is critical for its prevention and treatment. To address this need, numerous biological sensing platforms have been proposed for detection of the pathogenic biomarkers of the disease as a target molecule [1,2]. Amyloid-β (Aβ) peptides are known to be a representative clinical hallmark of AD and characterize its status [3,4]. Moreover, the structural deformation of Aβ into insoluble plaque forms, such as oligomers and fibrils, is a pathogenic event strongly associated with progression of the disease due to its neurotoxicity, which leads to cell death and neuronal dysfunction. Therefore, detection of Aβ and monitoring its condition as a target molecule can be a crucial strategy for early diagnosis of AD. To achieve this objective for clinical application, the biosensors must demonstrate an outstanding sensitivity, selectivity, and robustness, regardless of the working environment.

Over the last two decades, miniaturized cantilever sensors have attracted considerable attention for the development of biological and chemical sensing applications [5,6,7,8]. The detection of physical, chemical, and biological interactions on the cantilever surface relies on measuring changes in cantilever bending (static mode) or in the resonating characteristic (dynamic mode). 

In particular, the use of dynamic-mode cantilever sensors, one of the major operating methods, is suitable for detection of a small amounts of target molecules by measuring the shift of resonant frequency caused by effective mass loading on the surfaces compared to the static mode which observes the deflection of the beam caused by surface stress [9]. Most dynamic-mode cantilever sensors guarantee excellent behaviors in an air or under vacuum conditions. On the other hand, in a liquid environment, the sensing performance is drastically degraded by the reduction of the quality factor (Q-factor) on account of the viscous damping on the structure. Thus, a number of dynamic cantilever-based biosensors have been reported [10,11] and various technologies have also been proposed to overcome this issue, including a suspended microchannel resonator [12] and use of high-order resonance [13].

**Figure 1:**
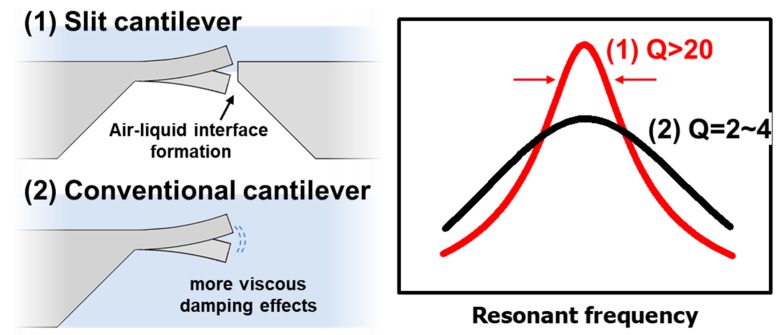
Schematic illustration of slit cantilever compared to the conventional structure of cantilever sensors in liquid environment and a conceptual diagram showing an enhanced quality-factor of the slit cantilever by reducing viscous damping due to formation of the air-liquid interface.

Here, we present a piezoelectric-driven microslit cantilever sensor (slit cantilever) for developing a biological application to diagnose AD in a liquid environment. The fabricated slit cantilever was resonated by an embedded PZT self-actuator to avoid the need for additional actuating modules. The resonant frequency the sensor was measured by a laser Doppler vibrometer (LDV). Only 2~3% error was shown in the air. The 5 μm gap of the slit surrounding the cantilever enabled a single side of the surface to be exposed when the liquid was introduced due to its meniscus. The Q-factor of the slit cantilever in liquid on the top surface showed a better value (Q = 35) in the case of filling both surfaces. It was sufficient to enable a high resolution in a liquid environment (Figure 1). 

To assess the slit cantilever as a biological sensing tool, the quantification and monitoring of the Aβ peptide self-aggregation process were studied using kinetic analysis. As noted above, a condition with a trace of Aβ is critical to the clinical diagnosis and pathological study of AD. Firstly, the quantitative detection of Aβ peptide with a concentration of 10 pg/mL to 1 μg/mL was performed. For the specific binding interactions of analytes, the specific-binding monoclonal antibody was chemically immobilized on the cantilever surface. The frequency change due to direct mass loading on the surface could be measured by the Aβ and antibody interaction. Furthermore, the dissociation constant (K_D_) of the Aβ-mAb binding affinity was calculated by fitting the quantification result to the thermodynamic isotherm. By using the slit cantilever to monitor the self-aggregation of monomeric Aβ peptide on the Aβ oligomeric intermediate, which was immobilized on the surface, the time-dependent responses caused by Aβ aggregation were investigated with different analyte concentrations. The self-growth of Aβ peptides on the functionalized surfaces was visually confirmed through topographic analysis.

## 2. Materials and Methods 

### 2.1. Sensor Fabrication

**Figure 2:**
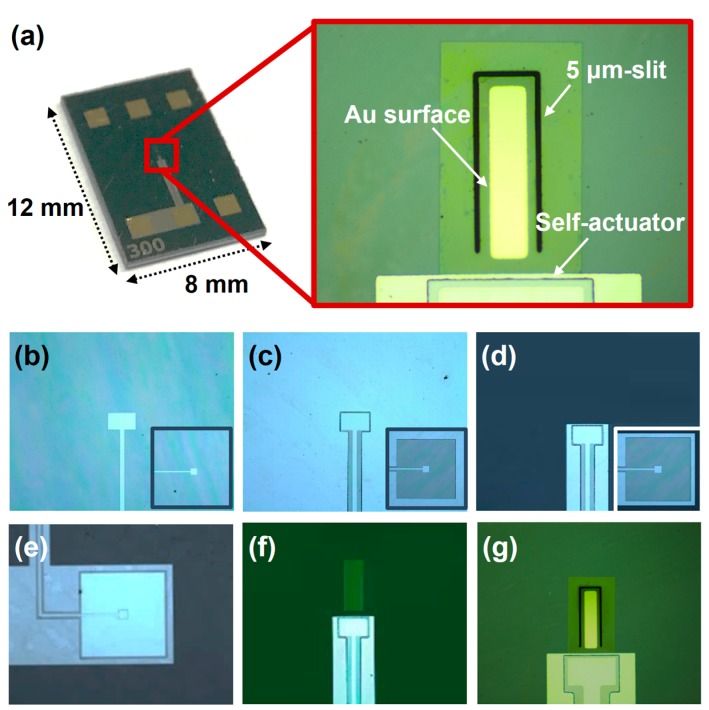
Optical images of the slit cantilever and fabrication process (**a**) a single die of the slit cantilever sensor and fabrication details for (**b**) upper Pt etching; (**c**) PZT wet-etching; (**d**) bottom Pt etching; (**e**) Au contact pad formation; (**f**) backside-Si bulk etching; and (**g**) biological active layer formation and cantilever releasing.

The microslit cantilever sensors were fabricated based on the fabrication processes used in previous studies [14,15]. However, as shown in Figure 2a, two major points were considered for a desirable operation in liquid: (1) the cantilever beam was released by a 5 µm gap of the slit to decrease viscous damping; (2) piezoelectric material, PZT, was removed from the cantilever structure and indirectly worked as an internal actuator. Details of the fabrication process and an optical image of the fabricated device are depicted in Figure 2b–g. First, a 4-inch multilayered SiN_x_/Si/SiN_x_/Ta/Pt substrate was prepared. A low-stress SiN_x_ thin film with a thickness of 1 µm was deposited on both sides of the Si wafer by low-pressure chemical vapor deposition. This formed the main structure. Next Ta and Pt thin films with respective thicknesses of 30 nm and 150 nm were deposited on the SiN_x_ followed by deposition of a PZT thin film with 2 µm thickness.

The PZT thin film was synthesized and deposited by a sol-gel method on Ta/Pt substrate. Then, the upper Pt thin film with a thickness of 100 nm was deposited on the PZT film by RF sputtering to form a Pt/PZT/Pt capacitance structure to function as a piezoelectric self-actuator. After the formation of multilayers on a 4-inch Si substrate, the top Pt, PZT, and bottom Ta/Pt layer were etched by an inductive coupled plasma (ICP) etching process (Figure 2a–c).

Then, a 200 SiO_2_ thin film was deposited and etched to function as a passivation layer, and Cr/Au patterns of 50/200 nm for contact pads were formed by a lift-off technique (Figure 2d). The Si was wet-etched with 30% potassium hydroxide (KOH) solution from the backside of the substrate (Figure 2e). Finally, the formation of a 10/50 nm-thick Cr/Au patterns as a functional area of biological interactions by a lift-off technique was followed by the releasing of the cantilever patterns by 5 µm gap surroundings (Figure 2g). As shown in the figure, the single cantilever has dimensions of 100 × 300 μm^2^ and a thickness of 1.25 μm.

### 2.2. Surface Functionalization

To detect the target molecules, a commercial monoclonal 6E10 antibody (mAb, 10 μg/mL, Covance, Princeton, NJ, USA) was immobilized on the cantilever surfaces by using a conventional coupling method. The mAb is known to include a specific binding ligand with the first 1–16 sequence of human Aβ peptides [16,17]. Prior to antibody immobilization, the slit cantilever with a 50 nm thick Au surface was cleaned with piranha solution (a 4:1 ratio of H_2_SO_4_ to H_2_O_2_) and rinsed with deionized water (DI). In addition, 10 mM of 11-Mercaptoundecanoic acid (11-MUC, Sigma-Aldrich, St. Louis, MO, USA) diluted in ethanol was treated for 12 h on the Au surface of the sensor to establish a self-assembled monolayer. The MUC-modified slit cantilever was immersed in a mixture of 1-ethyl-3-(3-dimethylaminopropyl) carbodiimide (EDC, 80 mM, Sigma-Aldrich) and N-hydroxysuccinimide (NHS, 20 mM, Sigma-Aldrich) dissolved for 2 h in 10 mM of phosphate-buffered solution (PBS, pH = 7.4). Then, mAb, which was diluted as 10 µg/mL in 10 mM PBS, was immobilized on the functionalized surface of the slit cantilever for 2 h, followed by rinsing with PBS and DI.

### 2.3. Preparation of Oligomeric Intermediates

The synthetic Aβ_42_ peptide (Sigma-Aldrich) was dissolved in dimethyl sulfoxide (DMSO) and diluted to a final concentration of 10 mg/mL in PBS solution. The solution was incubated without agitation at 37 °C for 48 h to form heterogeneous oligomeric intermediates [18,19,20]. The molecule acts as a seed for self-growth of Aβ peptides on the sensor surface. For monitoring the Aβ aggregation, the prepared oligomeric intermediates were immobilized on the cantilever surface. The slit sensor surface was activated with 11-MUC, followed by EDC-NHS coupling, which was the same as the mAb immobilization process. The functionalized slit cantilever was immersed in the prepared seed solution for 24 h. After rinsing with PBS, the sensor was treated with ethylenediamine (EDA, Sigma-Aldrich) to avoid nonspecific binding.

## 3. Results and Discussion

### 3.1. Resonant Frequency Characterization of the Slit Cantilever

**Figure 3:**
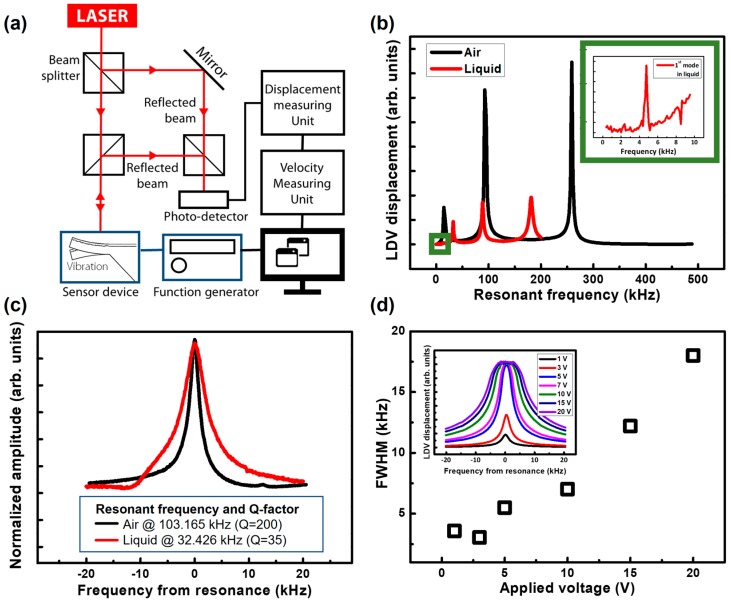
Characterization of resonating slit cantilever in air and liquid conditions. (**a**) An optical measurement setup of the slit cantilever sensor; (**b**) Measurement of first ~ third mode resonant frequencies in air and distilled water; (**c**) Direct comparison of an identical mode resonance peak between air and liquid phases; (**d**) Optimization of an induced voltage toward the PZT self-actuating layer.

To measure the resonant frequency of the slit cantilevers, the optical setup was established as depicted in Figure 3a. An external 3.0 V peak-to-peak voltage sinusoidal wave was induced at the top electrodes with 1.5 V of superimposed direct current (DC) voltage to vibrate the Pt-PZT-Pt capacitance and thereby enable measurement of the fundamental resonant frequency. Similar to most resonators, the slit cantilever has multiple vibrating modes. In this study, we measured the three dominant modes of resonant frequency within 500 kHz. As shown in Figure 3b (black line), the resonant frequencies of first to third modes were measured as 15.872 kHz, 103.165 kHz, and 289.426 kHz. According to Equation (1), the theoretical nth undamped resonant frequency of the cantilever in air (*f_n,air_*) can be calculated:

(1) $$f_{n,air}=\frac{\lambda_{n}^{2}}{2\pi }\sqrt{\frac{EI}{m^{*}L^{4}}}$$

where *λ_n_* is the eigenvalue of the nth vibrating mode, m* represents the distributed mass per unit length, *L* is the length of the cantilever beam, *E* denotes Young’s modulus, and *I* is the moment of inertia of the cross section.

Compared to theoretical values of vibrating modes, which were calculated by Equation (1), the difference was 2.1%, 1.5%, and 1.7% for first to third modes, respectively, and the wafer-level distribution was less than 5% (Table S1). The high similarity between the actual and theoretical values in the air condition indicates that our devices were well-fabricated, as intended. To inject the solution on the single surface of the sensor, the slit cantilevers were covered with a polydimethylsiloxane (PDMS) liquid cell, including a 500 μm width-straight microchannel with an inlet and outlet. It was assembled with a loading jig (Figure S1). 

As shown in Figure 3b (red line), the resonant frequency of all three modes measure at 4.7576 kHz, 32.426 kHz, and 88.266 kHz for first, second, and third modes, respectively, which decrease to nearly 30% of the initial values in air on account of the additional mass and viscosity of the filled solution. Although the Q-factor is also decreased, the second and third mode resonances show Q = 35 and Q = 21 have sufficiently high values to operate in a liquid environment as a sensing platform (Table 1).

According to a previous study [21], a fundamental resonant frequency in viscous environment can be calculated by Equation (2):

(2) $$f_{0,liq}=\frac{1}{2\pi }\sqrt{\frac{k_{0}+k_{\gamma }}{m_{0}}}$$

where *f*_0,*liq*_ is the dominant resonant frequency of the slit cantilever in a viscous environment, *k*_0_ denotes the spring constant of the cantilever, *k_γ_* represents the spring constant of the surface tension at the air–liquid interface, and *m*_0_ is the mass of the cantilever beam. 

Following Equation (2), the resonant frequency of the slit cantilever in the PBS solution was calculated 34.56 kHz, which indicated the second vibration mode of the slit cantilever. Figure 3c shows a direct comparison of a second mode resonance behavior of the slit cantilever, which has the highest Q-factor in both air and liquid environments. Finally, the operating stability of the slit cantilever was optimized with various conditions of the induced voltage to the PZT actuating layer. As shown in the inset of Figure 3d, the LDV displacements are increased and saturated when a higher voltage is induced. When more than 5 V is applied, however, the full width at half maximum value of the resonant frequency was increased by broadening the signals. Thus, the PZT self-actuator operating with 3 V has a stable resonance peak with the sharpest shape.

### 3.2. Quantitative Detection of Aβ Peptide

To evaluate the sensing performance of the slit cantilever in a liquid environment, quantification of the synthetic Aβ_42_ peptide, which has an identical sequence of 42-residue amino acids associated with human AD, was investigated in a physiological-level concentration. Prior to conducting biomolecular interactions, a drift of resonant frequency was verified and stabilized until steady state (Figure S2). The resonance responses reached its steady state within 200 min and the frequency maintained for 60 min with fluctuations up to 4 Hz which can be regarded as a noise level. Detection of target molecules with the slit cantilever is based on the capturing by immobilized mAb on the cantilever beam. It occurs in the measurement of the frequency shift due to mass loading on the surface. Firstly, the specific antibody (mAb) of 10 μg/mL was chemically immobilized on the 60 μm × 280 μm dimension of the Au surface (50 nm thickness) of the slit cantilever. The Aβ_42_ peptide was diluted with a ten-fold difference from 10 pg/mL to 1 μg/mL in 10 mM PBS. The prepared Aβ_42_ solution was individually introduced into the slit cantilever for 30 min, followed by rinsing with PBS. The changes of resonant frequencies in PBS were measured before and after the recognition interactions between mAb and target analytes. The resonant frequency decreased according to the concentration increment of target analytes owing to mass adsorption of Aβ_42_ to the specific binding site of the immobilized mAb. The average values of the frequency shifts were measured as −15.5, −56.5, −119.5, −472.5, −774 and −799 Hz for the concentrations from 10 pg/mL to 1 μg/mL, respectively. As noted earlier, these responses were satisfied with at least three-sigma significance in terms of the signal-to-noise ratio of the sensors.

**Figure 4:**
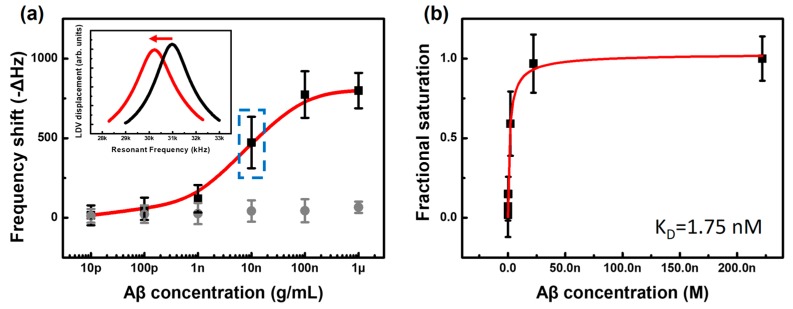
Quantification of Aβ_42_ peptide as a target analyte using the slit cantilever. (**a**) Responses of resonant frequency vs. logarithmic concentration of analytes (black square) and negative control with PSA protein (grey circle); (**b**) Estimation of the dissociation constant of specific recognition by fitting the thermodynamic isotherm model.

As shown in Figure 4a, a typical S-shape relationship of specific recognition reactions is shown with the saturated responses in the high concentration range Aβ (~1 μg/mL), which implies that the mAb binding sites are fully occupied by Aβ peptides. Compared to small frequency changes at the lowest level of analytes, an increment trend between the frequency shift in the range of 100 pg/mL to 100 ng/mL shows that the Aβ_42_ logarithmic concentration can be regarded as a dynamic range of the sensor with a sensitivity of 330 Hz/decade.

Based on the cerebrospinal fluid (CSF) levels of Aβ peptides in recent studies [22,23,24,25], the dynamic range of the slit cantilever suggested in the experimental results is highly effective when the AD subjects and normal subjects are distinguished. Moreover, when prostate cancer antigen (PSA) protein were introduced as a control experiment, no obvious responses were observed, as shown in Figure 4a (grey circle). 

The result of the Aβ_42_ quantification with the slit cantilever is plotted in the figure according to the relation between the fractional saturation of mAb and the concentration of target molecules on a linear scale (Figure 4b). The fractional value of saturation, which is an essential quantity for examining a binding affinity, is defined as the number of occupied binding sites on the mAb-immobilized surface divided by the total number of binding sites. We assumed the frequency response of 1 μg/mL reflects that the surface was fully saturated by the analytes to calculate the proportion. Using this approach, the equilibrium dissociation constant (K_D_) between the mAb and Aβ_42_ interactions could be calculated. Determining K_D_ is important for evaluating the relevance and standard protein analysis of the biosensors associated with the binding interactions between biological molecules, such as an antibody–antigen affinity or protein adsorption.

To obtain the above value, the simplest model of the 1:1 binding kinetic between target analytes (A) and mAb (B) was applied, as shown in Equation (3):

(3) $$\left[A]+[B]\overset{k_{on}}{\underset{k_{off}}{↔}}[\mathrm{AB}\right]$$

where *k_on_* is an association rate, and *k_off_* denotes the dissociation rate having different units: [M^−1^·s^−1^] and [s^−1^]. In the very simplest type of 1:1 binding affinity of Aβ_42_ (A) and mAb (B) to form a complex (AB), K_D_ is expressed as:

(4) $$K_{D}=\frac{k_{off}}{k_{on}}=\frac{\left[A][B\right]}{\left[\mathrm{AB}\right]}$$

According to Equation (4), the definition of K_D_ is manipulated to acquire the following equation:

(5) $$\frac{\left[\mathrm{AB}\right]}{\left[B\right]}=\frac{\left[A\right]}{K_{D}+[A]}$$

Meanwhile, Equation (5) predicts the hyperbolic plot of fractional saturation ([AB]/[B]) vs. Aβ concentration. This curve follows isotherms for relatively obscure thermodynamic reasons. Moreover, Equation (5) shows that the K_D_ value is defined as [A], where the fractional saturation meets 0.5. As shown in Figure 4b, the K_D_ value of 1.75 nM is obtained by a global fit of the Langmuir isotherm (χ^2^ = 0.010958), which was developed to describe the adsorption of molecules on a solid surface [26]. The acquired value is consistent with those of previous studies (1.2 nM) using conventional surface Plasmon resonance (SPR) [27]. The slit cantilever result demonstrates the validity of the specific recognition of Aβ peptides. It can thus be used as an analytic tool for biological kinetics.

### 3.3. Monitoring Time-Dependent Responses of Aβ Aggregation

The aggregation process of Aβ peptides on the cantilever surface was monitored by immobilization of oligomeric seeds on the cantilever surfaces. In terms of AD clinical research, it is important to quantitatively understand the self-assembly of the Aβ aggregates because neurotoxicity occurs during the growth process [28,29]. Compared to traditional methods for Aβ aggregation analysis, such as fluorescent assay or SPR, the Aβ growth rate can be measured within minutes and achieved in comparable concentrations of detection limits with the slit cantilever owing to its high sensitivity and direct quantification of bound analytes on the surface in solution [30,31]. Furthermore, in terms of detection techniques using a cantilever sensor, this approach using oligomeric seeds could be developed as a platform for the study of diseases-related biophysics following previous studies such as nanoparticles [32] or a single-stranded DNA probe [33].

**Figure 5:**
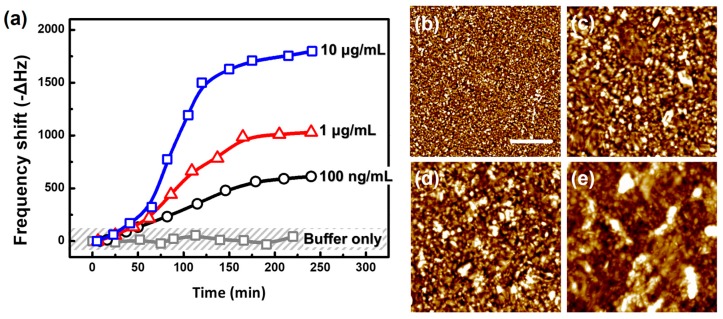
The resonant responses and topographic analysis of Aβ aggregation on sensor surfaces (**a**) Monitoring time-dependent responses of Aβ aggregation on the seed-immobilized cantilever surface with newly added Aβ_42_ peptide with concentrations of 100 ng/mL, 1 µg/mL, and 10 µg/mL; Topographic images of (**b**) the seed-immobilized Au surface and Aβ aggregation with concentration of (**c**) 100 ng/mL; (**d**) 1 µg/mL; and (**e**) 10 µg/mL. (A scale bar is 250 nm).

To lead Aβ growth, a heterogeneous oligomeric intermediate was prepared as the Aβ growth seed, and it was immobilized on the cantilever surface. Then, a monomeric Aβ_42_ solution with concentrations of 100 ng/mL (22.2 nM), 1 μg/mL (222 nM), and 10 μg/mL (2.22 μM) was injected into the sensor. The immobilized Aβ intermediates could be grown by the binding interactions of added Aβ peptides to form elongated aggregates [20,34]. The time-dependent responses of the resonant frequencies, which relied on quantitative amyloid growth on the sensor surface, were obtained as shown in Figure 5a. The results show the different growth rates and frequency shifts depending on the concentration of added Aβ_42_ peptide. 

When Aβ aggregation occurs, insoluble plaque forms accumulate. Therefore, several studies have reported the reaction following conventional binding kinetics [35,36,37]. We assumed the reaction on the slit cantilever followed an isotherm and fitted Langmuir isotherm to address the growth process. The association time constant was calculated as 0.007968 min^−1^ (125.5 min), 0.01010 min^−1^ (99.00 min), and 0.01130 min^−1^ (88.50 min) for the respective Aβ_42_ concentrations of 100 ng/mL, 1 μg/mL, and 10 μg/mL. Furthermore, the convergent values of the frequency changes at the saturated growth condition were estimated by fitting the isotherm as 813.0 Hz (100 ng/mL), 1132 Hz (1 μg/mL), and 1804 Hz (10 μg/mL) (Figure S3).

Aβ growth under identical circumstances was verified by topographic analysis using AFM. Figure 5b to e show the topologic images of Au surfaces with an Aβ aggregation reaction under different conditions. The sample surface roughness was 0.7341 nm, 1.413 nm, 2.224 nm, and 4.694 nm for the seed-only surface, and 100 ng/mL, 1 μg/mL, and 10 μg/mL for the monomeric Aβ peptide-added surface. When the oligomeric seeds were solely immobilized on the surface (Figure 5b), sub-nm-sized particles were distributed. However, oligomeric structures with length of tens of nanometers were observed when the monomeric Aβ_42_ peptide were introduced (Figure 5c,d). In particular, a significant size (>100 nm length) of aggregates was confirmed by AFM analysis with a concentration of 10 μg/mL Aβ_42_ peptide (Figure 5e). 

With these results, the Aβ aggregation process supported by prepared oligomeric seeds strongly relates to the initial concentration of newly added monomeric Aβ peptides. Thus, the proposed approach using the slit cantilever can have an important clinical application in AD diagnosis.

## 4. Conclusions

In this study, a cantilever sensor surrounded with a microslit structure was fabricated and its feasibility as a biosensing platform for AD diagnosis was evaluated. Firstly, the resonating characteristic of the slit cantilever was confirmed by measuring the resonant frequency, which was consistent with a theoretical calculation in both air and liquid conditions. In particular, the slit cantilever showed a better Q-factor (Q = 35) in the liquid phase than those of conventional cantilever sensors (Q = 2~4) since the single side of the sensor surface was in contact with the filled solution by the meniscus. Such properties of the slit cantilever enable study of the AD-related biophysical interactions in the liquid phase. A quantitative detection of Aβ_42_ peptides was conducted within the concentration of 100 pg/mL to 10 ng/mL. The results addressed both the valid dynamic range of an AD diagnosis and a dissociation constant (K_D_ = 1.75 nM) of the binding affinity between the specific mAb and the analytes. 

In addition, Aβ aggregation, a crucial pathogenesis in AD progression, was monitored with a different concentration of monomeric Aβ peptides added into the functionalized sensor surface. The time-dependent responses of resonant frequency indicated that an effective mass loading and a self-assembly growth rate on the sensor surface relied on an initial concentration of added Aβ peptides. The Aβ aggregation on the seed-immobilized surface was verified by a surface roughness comparison using topographic analysis. The results indicated that the proposed slit cantilever operated with an excellent sensing performance and overcame the major drawbacks of existing cantilever sensors in liquid. It is thus a promising technology for use in the clinical diagnosis of AD.

## Figures and Tables

**Table 1:** First ~ third resonance modes of resonant frequency in air and liquid environments.

Resonance Mode	Air		Liquid	
	Resonant Frequency	Q-Factor	Resonant Frequency	Q-Factor
1st	14.865 kHz	30	4.757 kHz	-
2nd	92.773 kHz	251	32.426 kHz	35
3rd	358.789 kHz	170	88.266 kHz	21

## Supplementary Materials

The following are available online at http://www.mdpi.com/1424-8220/17/8/1819/s1. Figure S1: Preparation of slit the cantilever for measuring the resonant frequency in a liquid environment with (a) a PDMS liquid cell and (b) assembly of a loading jig. Figure S2: Measuring resonant frequency shifts of slit cantilever in the liquid environment showing a drift effect with exposed time until steady state. Figure S3: Fitting the time-dependent responses to the thermodynamic isotherm in case of added Aβ_42_ concentration of (a) 100 ng/mL, (b) 1 µg/mL, and (c) 10 µg/mL added on slit cantilever. Table S1: Comparison of theoretical and actual values of first ~ third mode resonant frequency of slit cantilever.
