# Supplementary material for: Study of Alzheimer’s Disease-Related Biophysical Kinetics with a Microslit-Embedded Cantilever Sensor in a Liquid Environment
Source: Sensors (Basel). 2017 Aug 7;17(8):1819. doi: 10.3390/s17081819 (PMC5579575; doi:10.3390/s17081819)
Supplement: Supplementary file 1 [file sensors-17-01819-s001.pdf]

sensors

*Supplementary Information*

# Study of Alzheimer's Disease-Related Biophysical Kinetics with a Microslit-Embedded Cantilever Sensor in a Liquid Environment

**Myung-Sic Chae[1,2,†], Jinsik Kim[3,†], Yong Kyoung Yoo[4], Jeong Hoon Lee[4], Tae Geun Kim[2], Kyo Seon Hwang[1,*]**

1. Department of Clinical Pharmacology and Therapeutics, College of Medicine, Kyung Hee University, Seoul 02447, Republic of Korea
2. School of Electrical Engineering, Korea University, Seoul 02841, Republic of Korea
3. Department of Medical Biotechnology, College of Life Science and Biotechnology, Dongguk University, Seoul 10326, Republic of Korea
4. Department of Electrical Engineering, Kwangwoon University, Seoul 01897, Republic of Korea

† These authors contributed equally to this work.
* Correspondence: k.hwang@khu.ac.kr; Tel.: +82-2-958-2830

Academic Editor: name
Received: date; Accepted: date; Published: date

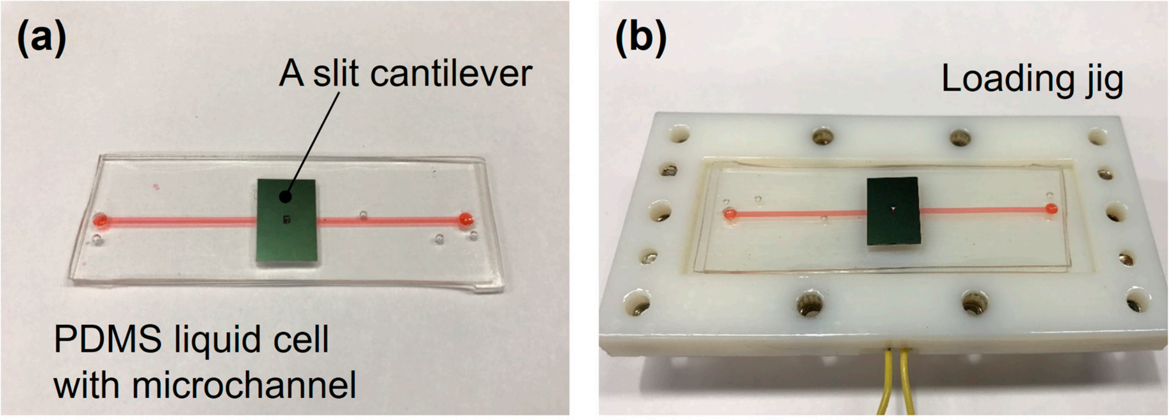


**Figure S1.** Preparation of slit the cantilever for measuring the resonant frequency in a liquid environment with (a) a PDMS liquid cell and (b) assembly of a loading jig

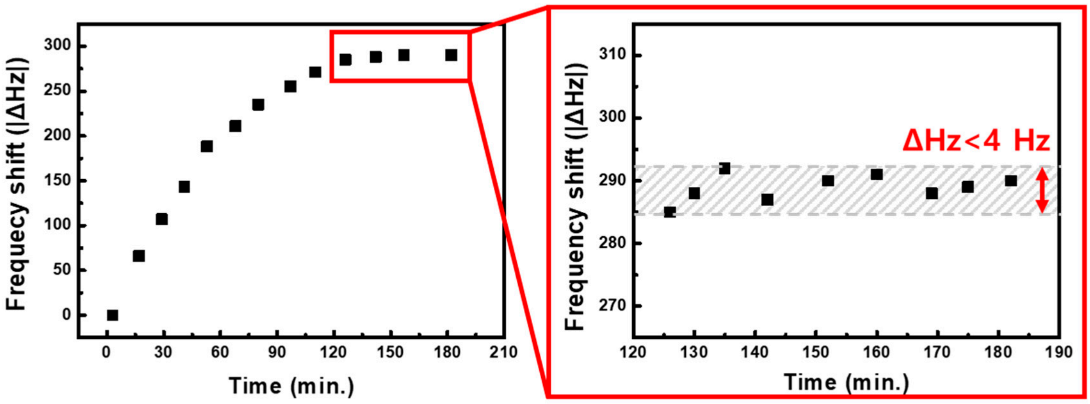


**Figure S2.** Measuring resonant frequency shifts of slit cantilever in the liquid environment showing a drift effect with exposed time until steady state.

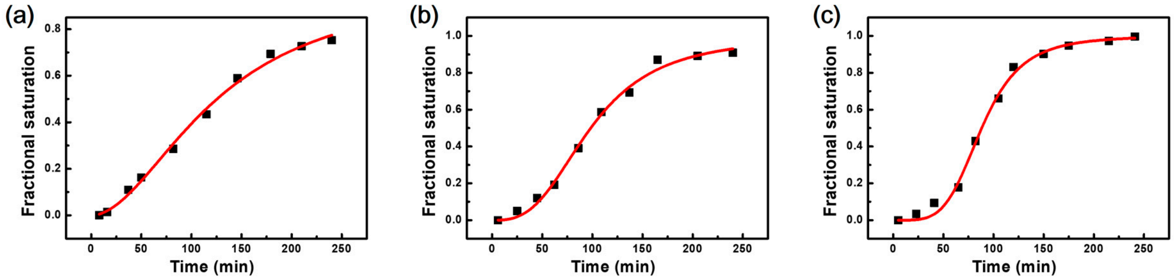


**Figure S3.** Fitting the time-dependent responses to the thermodynamic isotherm in case of added Aβ42 concentration of (a) 100 ng/mL, (b) 1 μg/mL, and (c) 10 μg/mL added on slit cantilever.

**Table S1.** Comparison of theoretical and actual values of 1st~3rd mode resonant frequency of the slit cantilever in air.

| Mode | Theoretical frequency | Measured frequency | Difference | C.V. in wafer-level |
|---|---|---|---|---|
| 1st | 16.209 kHz | 15.872 kHz | 2.1% | |
| 2nd | 101.588 kHz | 103.165 kHz | 1.5% | < 5% |
| 3rd | 284.480 kHz | 289.429 kHz | 1.7% | |
